# Rheumatoid Arthritis: The Stride from Research to Clinical Practice

**DOI:** 10.3390/ijms17060900

**Published:** 2016-06-08

**Authors:** Ill-Min Chung, Sarada Ketharnathan, Muthu Thiruvengadam, Govindasamy Rajakumar

**Affiliations:** 1 Department of Applied Bioscience, College of Life and Environmental Science, Konkuk University, Seoul 143-701, Korea; imcim@konkuk.ac.kr (I.-M.C.); thiruv30@yahoo.com (M.T.); 2 Department of Pathology, University of Otago, Dunedin 9016, New Zealand; sarada.biotech@gmail.com

**Keywords:** rheumatoid arthritis, HLA-DRB1, susceptibility genes, pharmacogenetics, miRNA

## Abstract

Over 70 different genetic variants with a significant association with rheumatoid arthritis (RA) have been discovered. Anti-citrullination protein antibodies (ACPA)-positive RA variants are more well-defined than their ACPA-negative counterparts. The human leukocyte antigen, HLA-DRB1 locus remains the prime suspect in anti-citrullination protein antibodies (ACPA)—positive RA. Different HLA-DRB1 alleles are linked to RA susceptibility across different ethnicities. With evolving techniques, like genome-wide association studies (GWAS) and single nucleotide polymorphism (SNP) arrays, more non-HLA susceptibility loci have been identified for both types of RA. However, the functional significance of only a handful of these variants is known. Their roles include increasing susceptibility to RA or in determining the speed at which the disease progresses. Additionally, a couple of variations are associated with protection from RA. Defining such clear-cut biological functions can aid in the clinical diagnosis and treatment of RA. Recent research has focused on the implication of microRNAs, with miR-146a widely studied. In addition to disease susceptibility, genetic variations that influence the efficacy and toxicity of anti-RA agents have also been identified. Polymorphisms in the *MTHFR* gene influence the effectiveness of methotrexate, the first line of therapy in RA. Larger studies are, however, needed to identify potential biomarkers for early disease identification and monitoring disease progression.

## 1. Introduction

Rheumatoid arthritis (RA) is an autoimmune disorder characterized by chronic synovial inflammation. Small joints of the hands and feet are more frequently affected; however, this varies with each individual. RA affects about 0.5%–1% of the population [1]. It is more common between the ages of 35 and 50 years, affecting three times more men than women [2,3].

RA rapidly progresses to cause joint deterioration and functional disability, eventually leading to unfavorable disease outcomes. Guidelines issued by an international task force of rheumatologists suggest that the primary target of RA treatment be clinical remission, defined as the absence of signs and symptoms of significant inflammatory disease [4]. Methods for early diagnosis and effective treatment are needed, as RA presents with a very narrow therapeutic window for remission. It has to be effectively managed within 3–6 months after the onset of the disease [5].

Identifying the etiology behind RA is critical in devising treatment and management strategies. Both genetic and environmental factors play significant roles in the pathophysiology of RA. Genetic factors contribute to about 60% of the risk for developing RA [6]. The relative risk of developing RA in first-degree relatives of affected individuals is about 2–4 [7,8].

## 2. Clinical Classification of Rheumatoid Arthritis (RA) Patients

RA is commonly classified into two subtypes: anti-citrullination protein antibodies (ACPA) positive and ACPA negative. Citrullination involves the conversion of the amino acid arginine into citrullin by the enzyme peptidylarginine deaminase (PAD), a process that is seen to occur during inflammation. About 75% of ACPA with undifferentiated arthritis will eventually develop RA within three years of follow-up [9]. ACPA-positive RA has a more aggressive clinical course compared to ACPA-negative RA. These two subclasses are essentially two genetically different diseases; however, the contribution of genetic factors appears to be equally high among both [10]. The ACPA-negative RA is a heterogeneous class, comprising within itself RA characterized by different antibodies, such as antibodies against carbamylated proteins (anti-CarP antibodies) [11].

## 3. Genes and RA

### 3.1. Human Leukocyte Antigen (HLA) Susceptibility Genes

The HLA locus is the most significant genetic contributor to RA, accounting for about 50% of the genetic susceptibility to RA [6]. Specifically, the HLA-DRB1 complex, a major histocompatibility (MHC) class II molecule involved in antigen presentation, is implicated. The HLA-DRB1 gene encodes a β chain protein which, when combined with the α chain, forms the HLA-DR antigen-binding heterodimer. Multiple RA-risk alleles within the HLA-DRB1 locus encode shared and conserved amino acid sequences at position 70–74: QKRAA, QRRAA, or RRRAA [12]. These sequences are present in the antigen-binding region of the β chain and are referred to as “shared epitopes” (SE). The mechanism by which the SE alleles contribute to the development of RA is not very clear. It has been postulated that the presence of these conserved sequences in the antigen-binding groove alters the way antigenic peptides are bound to and presented to T-cell lymphocytes. This, in turn, may trigger abnormal immune responses and lead to RA.

The HLA-DRB1*04 alleles and the HLA-DRB1*01 alleles typically share the conserved sequences. The presence of one HLA-DRB1 SE allele was associated with an odds ratio (OR) of 4.37 while the presence of two SE alleles was associated with an OR of 11.79, for RA. However, this association between SE alleles and RA was significant only in ACPA-positive individuals [13]. Furthermore, imputation studies on GWAS data have shown that the risk of RA associated with the HLA-DRB1 gene is strongest at the amino acid positions 11, 71, and 74 of the antigen-binding groove. With the most common haplotypes HLA-DRB1*1501 and HLA-DRB1*1502 as a reference, the highest ORs were associated with the presence of HLA-DRB*04 and HLA-DRB*10 subtypes. HLA-DRB*0401 was associated with an OR of 4.44 and HLA-DRB*0408, *0405, *0404, and *1001 with an OR of 4.22 each [14].

Individuals homozygous for the HLA-DRB1*0401 allele and individuals carrying two different shared epitope alleles are at a much higher risk of developing severe forms of RA. The HLA-DRB1 alleles mentioned in Table 1 have been significantly associated with RA, with the odds ratio varying across different ethnicities.

### 3.2. Non-HLA Susceptibility Genes

In addition to variations at the HLA locus, many other genes with implications in RA have been identified. Numerous candidate genes have been identified through techniques such as genome-wide association studies (GWAS) and single nucleotide polymorphism (SNP) array genotyping. Genes of importance in ACPA-positive RA include those encoding protein-arginine deiminase type 4 (*PADI4*), tyrosine-protein phosphatase non-receptor type 2 (*PTPN22*), cytotoxic T-lymphocyte protein 4 (*CTLA4*), interleukin-2 receptor subunit α (*IL2R* α), and signal transducer activator of transcription (*STAT4*) [15]. For ACPA-negative RA, the number of candidate genes identified so far is, comparatively, lesser and includes interferon regulatory factor 5 (*IRF5*) [16] and neuropeptide S receptor 1 (*NPSR1*) [17] gene polymorphisms.

Though numerous genetic associations with RA have been identified, only a handful has been studied for their biological function. About 80 different genetic loci have been associated with RA and many of them are also implicated in other autoimmune diseases, such as systemic sclerosis, lupus, myasthenia gravis, and Addison’s disease. Table 2 summarizes some of these recently identified loci and their importance in sub-typing RA for optimal treatment. The variations include non-synonymous polymorphisms located in coding or non-coding regions on the genome. While many of these variations increase the risk for RA susceptibility, some others have been useful markers in monitoring disease prognosis.

## 4. Functional Role of Non-HLA Genetic Variations

Genetic polymorphisms may exert their influence by altering transcriptional activity, splicing machinery, mRNA or protein stability, and translational and post-translational modifications. The most widely researched RA polymorphism to date is rs2476601. This is a non-synonymous polymorphism: Arg620Trp in the *PTPN22* gene, which downregulates T-cell receptor (TCR) signaling by dephosphorylation of certain kinases. The Arg620Trp variant results in a loss of function allele that causes increased proliferation, activation and thymic selection of T-cells. Dendritic cells and B-cell activation are also increased [28]. Another important genetic factor is the *PADI4* gene, encoding the enzyme that is responsible for the process of citrullination, as described earlier. Variations in the gene increase the production of citrullinated proteins leading to increased interaction with the HLA-DRB1 SE molecules [29]. Thus, these autoantigens elicit an adaptive immune response, progressing to RA [30].

Surface protein expression levels in immune cells, such as monocytes, CD4^+^ naïve T-cells, and memory cells, are influenced by polymorphisms present in the *IL2RA* gene. Changes in expression translate to changes in the thresholds of stimuli needed for activation of these cells [31]. T-cell activation requires two different signals: the first, an antigen-specific interaction, and the second, signals from co-stimulatory molecules. *CTLA4*, also called *CD152*, is a protein receptor expressed on activated T-cells that down-regulates T-cell function. It acts as a checkpoint in regulating self-tolerance and susceptibility to autoimmune diseases [32]. The *STAT4* gene encodes a transcription factor that regulates the expression of genes responsible for maturation of T-cells. Through the JAK/STAT pathway, *STAT4* relays signals initiated by interleukin-12, interleukin-23, and type I interferons, regulating Th1 and Th17 cell responses [33]. Both of these T-cell types play critical roles in autoimmune diseases and are key regulators of RA pathogenesis in humans.

*CCR6* encodes a chemokine receptor localized on the surface of immature dendritic cells and memory T-cells, and binds the MIP-3A (macrophage inflammatory protein 3-α) ligand. CCR6^+^ T_h_ cells are potent inducers of synovial inflammation. These cells trigger off an inflammatory cycle, assisted by IL-17A and TNF-α. This results in the production of interleukins IL-1β, IL-6, IL-8, prostaglandins PGEs, and matrix metalloproteinases (MMPs) by synovial fibroblasts [34]. CCR6^+^ T_h_ cells are, therefore, representative of RA with a worse prognosis. DNMT3B, a DNA methyltransferase, catalyzes methylation of unmodified CpG islands, *de novo*. A polymorphism in the *DNMT3B* gene, −C283T, has been shown to decrease promoter activity of the gene. Patients carrying the variant allele have a greater propensity for rapid joint destruction than others. Synovial inflammation may be caused by an upregulation of the extent of gene-specific demethylation within the affected cells [26]. Epigenetic forces may regulate the expression of various cytokines that may, in turn, facilitate synovial inflammation and disease.

*TNFAIP3*, also called A20, is a deubiquitinating protein that can inhibit the function of a critical transcription factor, NF-κB and, hence, regulate expression of all downstream genes. Aberrant A20 expression is related to polyarthritis that has similar features to RA in mice [35]. The reported polymorphism rs2230926 results in a non-synonymous amino acid change Phe127Cys. The variant allele causes reduced downregulation of NF-κB, leading to excessive immune reactions and enhanced autoimmune reactivity [36].

## 5. RA Protective Genes

Certain genetic factors, unlike the ones mentioned above, confer a protective trait against RA. Among the HLA-DRB1 alleles only the HLA-DRB1*13:01 allele has been associated with a protective phenotype. The amino acid sequence corresponding to this allele at positions 70–74 is DERAA. Earlier studies had shown that all HLA-DRB1 alleles with the DERAA sequence confer protection against RA. Some studies also suggested that the presence of certain other amino acid sequences at defined positions is associated with RA protection; for example, the presence of isoleucine at position 67 [37]. Such controversies were, however, resolved by a meta-analysis study performed on about 3000 European patients from the following cohorts: the European Research on Incapacitating Diseases and Social Support (EURIDISS), the Swedish Epidemiological Investigation of RA (EIRA), the Leiden Early Arthritis Clinic (EAC) and the BehandelStrategieen (BeST) trial [38]. The study concluded that only the HLA-DRB1*13 alleles and, more specifically, the HLA-DRB1*13:01 alleles is associated with protection. This association is, however, valid for only ACPA-positive RA. No such protective alleles have been identified in ACPA-negative RA [10]. HLA-DRB1 molecules that contain the DERAA sequence have been proved to be protective when they occur as non-inherited maternal antigens [39], a phenomenon similar to Rhesus positive RhD genotype inheritance.

## 6. MicroRNAs in Rheumatoid Arthritis

MicroRNAs are small non-coding RNA molecules that negatively regulate the expression of genes. A number of inflammatory cytokines, including TNF-α and interleukin-1β, are actively involved in RA pathogenesis. This may be so by the stimulation of expression of certain miRNAs, like miR-146a and miR-155, in monocytes and macrophages. RA patients have also shown high levels of miR-132 and miR-16, expressed by these two cell populations. Despite high expression of miR-146a, its targets *TRAF6* and *IRAK-1* failed to be aberrantly expressed in RA patients [40,41]. This indicates that, in RA pathogenesis, regulation of *TRAF6* and *IRAK-1* genes is lost, facilitating the prolonged production of TNF-α.

MicroRNA-146a expression levels in the peripheral blood of RA patients were comparable to the levels seen in synovial tissue and fibroblastic cells [40]. However, as elevated miR-146a is seen in diseases besides RA, such as osteoarthritis, its use as a diagnostic biomarker is questionable. Nevertheless, it can be used to monitor the disease course in RA patients. It would be useful to investigate if polymorphisms and other genetic variations in the miR-146a target genes could prove to be useful for the diagnosis of RA. High expression of miR-155 was seen in synovial tissue of RA patients. This expression correlated well with the repression of MMPs [42]. The role of miR-124 in regulating cyclic-dependent kinase-2 (CDK-2) and monocyte chemotactic protein-1 (MCP-1) is dysregulated in RA [43]. Two studies have reported associations between suppression of microRNAs and RA pathogenesis: miR-363 and miR-498 were downregulated in CD4^+^ T-cells [44], and miR-124a in synovial fibroblasts [43]. Other miRNAs (Table 3) that play significant roles in RA pathogenesis [45] include miR-223, miR-203, miR-363, and miR-498. The major epigenetic forces that are operative in RA are DNA hypomethylation and histone hyperacetylation. Both of these mechanisms lead to enhanced synovial proliferation, leading to arthritis. Differential expression of other types of RNA, such as long non-coding RNA, has been observed in patients with rheumatoid arthritis. Long non-coding RNA growth arrest-specific 5 (GAS5) [46], LOC100652951, and LOC100506036 [47] regulates immune functions in RA.

## 7. Personalized Treatment for RA

The genetic make-up of an individual drives how he/she responds to drugs. Genetic variations may correlate with enhanced efficacy of the drug or may make the individual highly susceptible to adverse drug reactions. Among the disease-modifying anti-rheumatic drugs (DMARDs), methotrexate (MTX) forms the first line of treatment. It exerts its anti-inflammatory effects by inhibiting enzymes involved in the folate and adenosine pathways. A number of genetic polymorphisms influence MTX efficacy and toxicity in RA. Some of the genes implicated are *MTHFR*, *DHFR*, *ABCB1*, *SHMT*, *TYMS*, and *RFC1* [48,49,50,51,52]. The *MTHFR* gene has been widely studied, and two SNPs have been identified to have significant effects on MTX efficacy and toxicity, but the results have not been consistent [53]. Polymorphisms *MTHFR* C677T and *MTHFR* A1298C diminish the activity of the MTHFR enzyme, thereby disrupting the folate pathway. The two SNPs may also act in synergy. Mutations in *ATIC* and *TYMS* have been correlated with increased efficacy but increased toxicity with MTX treatment. Increased efficacy with hydroxychloroquine is conferred by mutations in the *IL10* or the *TNF* genes. Genetic variations in the *TPMT* gene render individuals at high risk to azothioprine toxicity.

As TNF-α predominantly governs RA pathogenesis, TNF antagonists such as infliximab have been very effective in treating RA. However, about 30% of RA patients fail to respond to this therapy, and among the others, the response varies from almost complete remission to disease progression of clinical symptoms [54]. An SNP in the promoter region of the TNF gene, −A308G showed an association with the TNF inhibitor etanercept. Individuals carrying the variant allele exhibited better therapeutic response [55]. This association was also seen with infliximab. The adverse effects seen with DMARDs usage are a lot of serious and opportunistic infections. Patients treated with anti-TNF antibody therapy had a high risk of infections with an odds ratio of 2 [56]. The polymorphisms *TNF* − G238A, *LTA* + G365C, and *FCGR3A* + Phe176Val were significantly associated with the development of urinary tract infections when subjected to treatment with MTX and etanercept. An additive effect was seen with the number of risk alleles proportional to the level of risk [57]. A variation in the *FCGR* gene, Val58Phe, has been strongly associated with rituximab efficacy.

Although numerous genetic variants with pharmacogenetic potential [58] have been identified (Table 4), there lacks a consistency in the phenotype conferred. Additionally, it is essential to sieve out from these numerous variants the ones that can serve as early predictors of RA and enable disease monitoring.

## 8. Conclusions

A large amount of data on the genetic etiology of RA is available. We have not attained a stage where genetic predisposition of this disease can be accurately used in clinical practice. This is because we need to gain more understanding about the biological functions of the various genetic factors, so as to select the most significant one. Additionally, we need to elucidate the contribution of environmental factors to RA in addition to the complexities of phenomena such as epigenetics. Only then, an accurate prediction model can be designed. There lacks ethnicity-specific information, with respect to the genetics of RA. This is critical because the variants that influence RA in one population type have almost a null effect on other population types. Genetic testing can revolutionize the treatment of RA by predicting predisposition and treatment response, thereby enabling physicians to achieve enhanced remission rates.

## Figures and Tables

**Table 1:** Clinical significance of human leukocyte antigen HLA-DRB1 alleles in rheumatoid arthritis (RA).

Genotype HLA-DRB1	Amino Acid Sequence (70–74)	Disease Severity
*0101	QRRAA	Intermediate
*0102	QRRAA	Intermediate
*0401	QKRAA	Severe
*0404	QRRAA	Intermediate
*0405	QRRAA	Intermediate
*0408	QRRAA	Intermediate
*1001	RRRAA	Intermediate
*1301	DERAA	Protective
*1402	QRRAA	Intermediate

**Table 2:** Clinical significance of recently identified susceptibility loci in RA.

Chromosome	Candidate Gene	Variations	Phenotype	References
1	*PTPN22*	rs2476601	Anti-citrullination protein antibody (ACPA) positive RA with worse prognosis	[18]
1	*IL6R*	rs8192284	Influences disease activity of ACPA-positive RA	[19]
2	*SPRED2*	rs934734	Increased risk for ACPA-positive RA	[20]
3	*PXK*	rs13315591	Increased risk for ACPA-positive RA	[20]
4	*RBPJ*	rs874040	Increased risk for ACPA-positive RA	[20]
5	*ANKRD55*	rs6859219	Increased risk for ACPA-positive RA	[20]
6	*CCR6*	rs3093023	Increased RA susceptibility	[21]
6	*TNFAIP3*	rs675520 rs9376293	Increased rate of joint destruction in ACPA-positive RA	[22]
6	*TAGAP*	rs629326	Increased risk for ACPA-positive RA	[23]
7	*IRF5*	rs10488631	Increased risk for ACPA-positive RA	[20]
15	*RASGRP1*	rs8043085	Increased risk for ACPA-positive RA	[23]
17	*CCL3*	Undefined	Increased RA susceptibility	[24]
19	*CARD8*	C10X	Worse disease course in early RA	[25]
20	*DNMT3B*	−283C/T	Influences progression of joint destruction in RA	[26]
20	*CD40*	rs6032662	Increased risk for ACPA-positive RA	[23]
22	*GATSL3*	rs1043099	Increased RA susceptibility	[27]
X	*IRAK1*	rs13397	Increased RA susceptibility	[23]

**Table 3:** Aberrant micro-RNA expression in RA.

Source	Upregulated miRNA	Downregulated miRNA
CD4^+^ naïve T-cells	miR-223, miR-146a	miR-363, miR-498
PBMCs	miR-146a, miR-155, miR-132, miR-16	
Synovial fibroblasts	miR-203, miR-155	
Synovial tissue	miR-146a, miR-155	
Joint fluid	miR-146a, miR-155, miR-223, miR-16	
RA fibroblast-like synoviocytes	miR-346	miR-124a

**Table 4:** The pharmacogenetics of RA.

Drug	Gene	Variant	Phenotype
Methotrexate	*SLC19A1* (*RFC-1*)	G80A	Increased or unaffected efficacy
	*MTHFR*	C677T	Increased toxicity
	*MTHFR*	A1298C	Controversy regarding toxicity and efficacy
	*ATIC*	C347G	Increased gastrointestinal toxicity; increased efficacy
	*ABCB1* (*MDR1*)	C3435T	Controversy regarding toxicity and efficacy
	*MTHFD1*	G1985A	Decreased efficacy
	*SHMT1*	C1420T	Increased toxicity
	*TYMS*	5′-UTR repeat element	Decreased efficacy
Hydroxychloroquine	*IL-10*	A308G	Increased efficacy
	*IL-10*	A1082G	Increased efficacy
Azathioprine	*TPMT*	TPMT*2, *3A, *3C	Increased toxicity
	*ITPA*	C94A	Increased toxicity
Anti-TNF agents	*TNF*	G308A	Increased efficacy in most studies; effect on toxicity is controversial
	*TNF*	A238G	Increased efficacy
	*TNFRSF1B*	T196G	Decreased or no effect on efficacy
	*FCGR3A*	Val158Phe	No effect on efficacy
	*PTPRC*	SNP	Increased efficacy
	*MAPK14*	SNP	Increased efficacy of anti-TNF antibodies (infliximab, adalimumab)
Rituximab	*FCGR3A*	Val158Phe	Increased efficacy or no effect
